# Asymbiotic germination of *Vanilla planifolia* in relation to the timing of seed collection and seed pretreatments

**DOI:** 10.1186/s40529-021-00311-y

**Published:** 2021-05-03

**Authors:** Chih-Hsin Yeh, Kai-Yi Chen, Yung-I. Lee

**Affiliations:** 1 Taoyuan District Agricultural Research and Extension Station, Council of Agriculture, Executive Yuan, Taoyuan, 327 Taiwan, ROC; 2 Department of Agronomy, National Taiwan University, Taipei, Taiwan, ROC; 3 Biology Department, National Museum of Natural Science, 40453 Taichung, Taiwan, ROC; 4 Department of Life Sciences, National Chung Hsing University, 40227 Taichung, Taiwan, ROC

**Keywords:** Micropropagation, Embryo, Seed coat, Seed pretreatment, *Vanilla*

## Abstract

**Background:**

*Vanilla planifolia* is an important tropical orchid for production of natural vanilla flavor. Traditionally, *V. planifolia* is propagated by stem cuttings, which produces identical genotype that are sensitive to virulent pathogens. However, propagation with seed germination of *V. planifolia* is intricate and unstable because the seed coat is extremely hard with strong hydrophobic nature. A better understanding of seed development, especially the formation of impermeable seed coat would provide insights into seed propagation and conservation of genetic resources of *Vanilla*.

**Results:**

We found that soaking mature seeds in 4% sodium hypochlorite solution from 75 to 90 min significantly increased germination. For the culture of immature seeds, the seed collection at 45 days after pollination (DAP) had the highest germination percentage. We then investigated the anatomical features during seed development that associated with the effect of seed pretreatment on raising seed germination percentage. The 45-DAP immature seeds have developed globular embryos and the thickened non-lignified cell wall at the outermost layer of the outer seed coat. Seeds at 60 DAP and subsequent stages germinated poorly. As the seed approached maturity, the cell wall of the outermost layer of the outer seed coat became lignified and finally compressed into a thick envelope at maturity. On toluidine blue O staining, the wall of outer seed coat stained greenish blue, indicating the presence of phenolic compounds. As well, on Nile red staining, a cuticular substance was detected in the surface wall of the embryo proper and the innermost wall of the inner seed coat.

**Conclusion:**

We report a reliable protocol for seed pretreatment of mature seeds and for immature seeds culture based on a defined time schedule of *V. plantifolia* seed development. The window for successful germination of culturing immature seed was short. The quick accumulation of lignin, phenolics and/or phytomelanins in the seed coat may seriously inhibit seed germination after 45 DAP. As seeds matured, the thickened and lignified seed coat formed an impermeable envelope surrounding the embryo, which may play an important role in inducing dormancy. Further studies covering different maturity of green capsules are required to understand the optimal seed maturity and germination of seeds.

## Background

Vanilla planifolia is a vanilla orchid native to Mexico and Central America (Bory et al. 2008), and has been planted in many tropical regions around the world to produce natural vanilla flavor (Sreedhar et al. 2007). Since the mature seeds of V. planifolia hardly germinate, V. planifolia is usually propagated commercially by vegetative propagation methods, such as cutting stem or callus culture regeneration (Palama et al. 2010; Havkin‐Frenkel and Belanger 2018; Lee 2018). Nevertheless, seed propagation can produce offspring with differing genotypes that is important to generate novel traits in breeding programs. Asymbiotic seed germination technique has been widely used for the commercial propagation of many orchids (Knudson 1922; Yam and Arditti 2017). However, the application of this technique to some temperate terrestrial orchids is complicated, e.g. Cymbidium, Cypripedium, Epipactis (Kako 1976; Van der Kinderen 1987; Rasmussen 1995; Miyoshi and Mii 1998). In these intricate-to-germinate orchids, the seeds have deep dormancy and the germination of mature seeds could be extremely low in a range from 0 to 5 %. Causes of low germination of some orchids may relate to the impermeability of the seed coat during seed maturation (Lee et al. 2005) or the accumulation of inhibitory substances to germination (Van Waes and Debergh 1986; Van der Kinderen 1987; Lee et al. 2007, 2015). Although Vanilla is pantropical in distribution, seed germination in vitro of V. planifolia is still considered intricate (Knudson 1950).

Successful asymbiotic germination may be related to the timing of seed collection (Arditti 1967; Lee et al. 2005), components of culture medium, such as organic nutrients, carbon sources, plant growth regulators (Lo et al. 2004; Gayatri and Kavyashree 2005; Dutra et al 2008), light intensity and temperature ranges (Knudson 1950; Suzuki et al. 2012), and seed pretreatments (Lee 2011). Like most orchids, *V. planifolia* produces many seeds that contain globular embryos without the endosperm (Clements and Molvray 1999). The seed coat of *V. planifolia* is black and hard, which contrasts markedly with the thin transparent seed coat of most orchid seeds (Cameron and Chase 1998; Nishimura and Yukawa 2010). Mature seeds of *V. planifolia* usually have very low germination (Knudson 1950; Menchaca et al. 2011), which might be due to the impermeability of the hardened seed coat (Withner 1955; Lee et al. 2005, 2007, 2015; Van der Kinderen 1987; Van Waes and Debergh 1986). Despite the low germination of mature seeds, there are some practical advantages of using mature seeds for propagation, e.g. the long term storage and the long distance shipment (Steele 1996). Therefore, it is necessary to find out the pretreatment conditions for breaking mature seed dormancy of *V. planifolia*.

This study aimed to establish an efficient propagation method of *V. planifolia* via asymbiotic germination. We first tested the effect of seed maturity on asymbiotic germination and determined the optimal timing for culturing immature seeds. In order to improve the germination of mature seeds, we examined the effect of different combinations of sodium hypochlorite concentrations and soaking durations on germination. We also documented the morphological, histological, and histochemical changes of seed development within a defined timescale, from fertilization to seed maturity. Such information would provide insights into mass propagation to meet commercial needs and breeding programs for vanilla production.

## Methods

### Plant material and seed collection

**Fig. 1:**
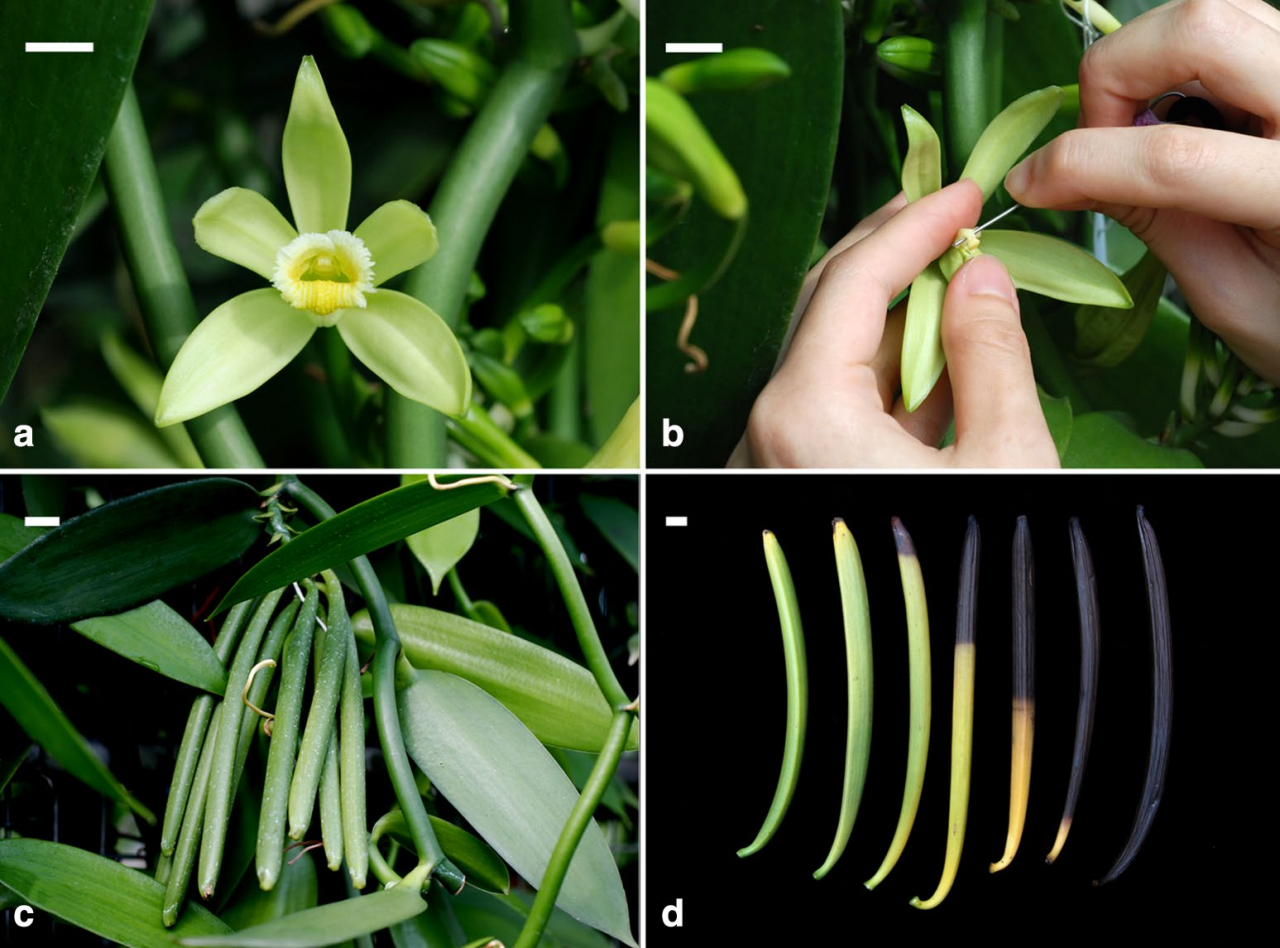
Flowers and developing pods of *V. planifolia*. **a** Flower at the time of anthesis. Scale bar = 1 cm. **b** Flower was hand-pollinated by transferring the pollinia onto the stigma of the same flower. Scale bar = 1 cm. **c** Developing pods at 60 DAP. Scale bar = 1 cm. **d** Pods turned yellow by 240 DAP and became black by 300 DAP. Scale bar = 1 cm

The mature plants of *V. planifolia* were maintained in a greenhouse at Taoyuan District Agricultural Research and Extension Station at Taoyuan City, Taiwan. Anthesis generally occurs in late April each year (Fig. 1a). For the pod setting, flowers were hand-pollinated by transferring the pollinia onto the stigma of the same flower (Fig. 1b). Developing pods (Fig. 1c) were collected at regular intervals for morphological measurement, histology, and seed germination experiments. In January the next year, pods began to mature and turned yellow (Fig. 1d). In each experiment, seeds were collected at least from three pods at regular intervals after pollination.

### Evaluation of asymbiotic germination percentage

The pods of different developmental stages were surface-sterilized with a 1.8% sodium hypochlorite solution with one drop of a wetting agent (Tween 20, Sigma-Aldrich) for 15 min. After surface sterilization, the capsules were cut open, and seeds were scooped out with forceps onto the culture medium. To ensure the seed quality and developmental stages of each capsule, the remaining seeds of each capsule were fixed and examined under a microscope. The culture medium used was 1/2 Murashige and Skoog (MS) (Murashige and Skoog 1962) supplemented with 2 mg l^–1^ glycine, 0.5 mg l^–1^ niacin, 0.5 mg l^–1^ pyridoxine HCl, 0.1 mg l^–1^ thiamine, 1 g l^−1^tryptone, 20 g l^−1^ sucrose, and solidified with 7 g l^−1^ agar (plant cell culture, tested, powder; all Sigma-Aldrich). The pH value was adjusted to 5.7 before autoclaving at 121 °C and 1.2 kg cm^2^ for 20 min. An amount of 10 ml medium was placed into each culture tube (20 × 100 mm). There were ca. 100 seeds per culture tube. After sowing, the cultures were incubated in a growth room under a 16/8 h photoperiod with daylight fluorescent lamps (20 W, China Electric, Taipei) at light intensity 30 µmol m^−2^ s^−1^. Germination percentage was recorded 60 days after sowing.

### Effect of seed pretreatments on germination

To examine the effectiveness of different concentrations and durations of sodium hypochlorite pretreatments in improving germination, mature seeds were collected from capsules at 135 DAP. There were four capsules collected for each pretreatment. Mature seeds were scooped into test tubes, then filled with 30 ml sodium hypochlorite solution. The pretreatments included soaking the seeds in 0.5, 1, 2 or 4% sodium hypochlorite solution with one drop of Tween 20, for 15, 30, 45, 60, 75 or 90 min. In control, seeds were soaked only in water. After pretreatments, seeds were washed three times with sterilized distilled water, then inoculated on 1/2 MS medium as described above. There were ca. 300 seeds per culture tube. The culture conditions and germination record were same as asymbiotic germination procedure as described above.

### Histological and histochemical studies

Developing seeds were collected and fixed in 2.5% glutaraldehyde and 1.6% paraformaldehyde buffered with 0.1 M phosphate buffer (pH 6.8) for 48 h at room temperature. Seeds were then dehydrated in an ethanol series, then infiltrated gradually (3:1, 1:1, and 1:3 100% ethanol: Technovit 7100, 24 h each) by using Technovit 7100 resin (Kulzer & Co., Germany), followed by three changes of pure resin. Seeds were then embedded in resin, as described by Yeung and Chan (2015). Sections of 3-μm thick were cut using a Reichert-Jung 2040 Autocut rotary microtome. These sections were stained with periodic acid–Schiff (PAS) procedure for structural carbohydrates and counterstained with 1% (w/v) amido black 10B in 7% acetic acid for protein (Sigma-Aldrich, St. Louis, MO, USA) or 0.05% (w/v) toluidine blue O (TBO, Sigma-Aldrich) for general histological staining (Yeung, 1984). For detecting the deposition of cuticular material in developing seeds, sections were stained with 1 μg ml^−1^ Nile red (Sigma-Aldrich) for 1 min, then washed in running tap water for 3 min. The fluorescence pattern of Nile red was viewed under an epifluorescence microscope (Axioskop 2, Carl Zeiss AG, Germany) equipped with the Zeiss filter set 15 (546/12 nm excitation and 590 emission). All images were recorded by using a CCD camera attached to the microscope.

### Rooting and acclimatization of in vitro seedlings

After 150 days of culture, developing protocorms with roots were transferred onto seedling growth medium: 1/2 MS medium supplemented with 20 g l^–1^ sucrose, 1 g l^–1^ activated charcoal powder (C9157, Sigma-Aldrich), 20 g l^–1^ potato homogenate, and 7 g l^–1^ agar for growing seedlings described by Lee (2011). The potato was boiled for 10 min, then peeled and cut into ca. 1-cm^3^ cubes, then homogenized with a kitchen blender. The pH of the medium was adjusted to 5.6 before autoclaving at 121 °C for 20 min. An amount of 100 ml medium was dispensed into a 500-mL culture flask. After transferring to the seedling growth medium, flasks were placed in a growth room under a 16/8 h photoperiod with daylight fluorescent lamps (20 W, China Electric, Taipei) at light intensity 30 µmol m^−2^ s^−1^. After 90 days of culture in the seedling growth medium, seedlings of about 10 cm tall with 4 leaves were taken out of flasks.

### Experimental design and statistical analysis

All experiments were performed in a completely randomized design and repeated three times; 12 replicates (culture tubes) were used for each treatment, with one explant planted in each plate. Data were analyzed by using analysis of variance (ANOVA) with Fisher’s protected least significant difference test at *P* < 0.05. All data were analyzed with SAS v9.0 (Cary, NC, USA).

## Results

### Seed germination can be achieved using asymbiotic germination technique at early seed developmental stages of *V. planifolia*

**Fig. 2:**
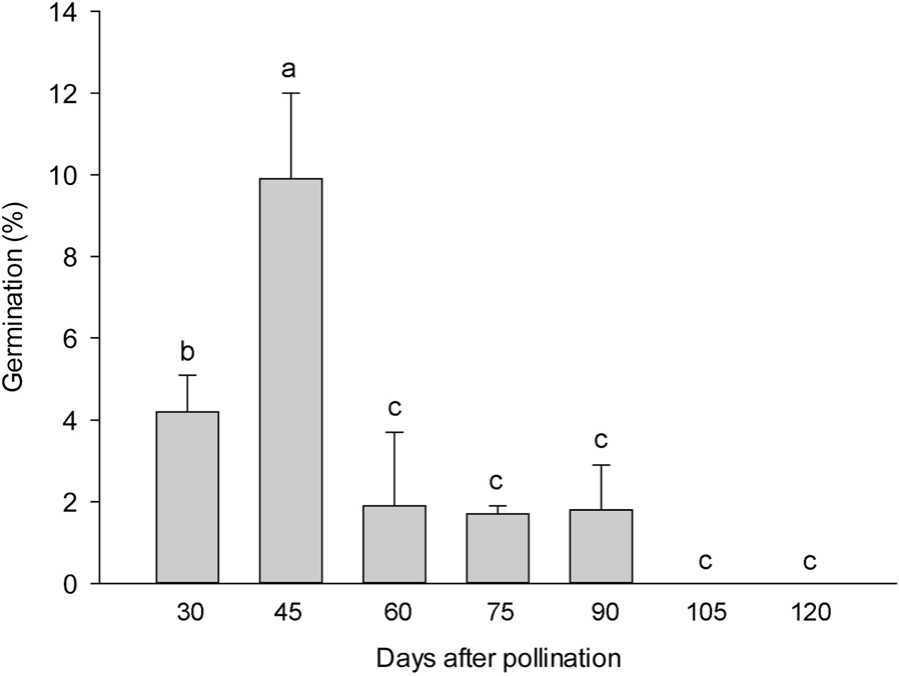
Mean percent germination of *V. planifolia* seeds at each successive 15 days after pollination on 1/2 MS medium. Data were scored after 60 days of culture. Data are mean ± SD (n = 10)

In this study, different stages of developing seeds were collected in an interval of 15 days from the day of fertilization to 300 DAP, and their germination percentages under the asymbiotic germination treatments were investigated. Seed germination occurred after 30 DAP (Fig. 2). The seed germination percentage reached 9.9% at 45 DAP, the highest germination percentage through the whole experiment. The germination percentage then markedly declined to approximately 2% at 60 DAP. This low germination percentage lasted to 90 DAP. Almost no seed germination was observed after 120 DAP up to 300 DAP (data not shown).

### Effect of seed pretreatments on germination

**Fig. 3:**
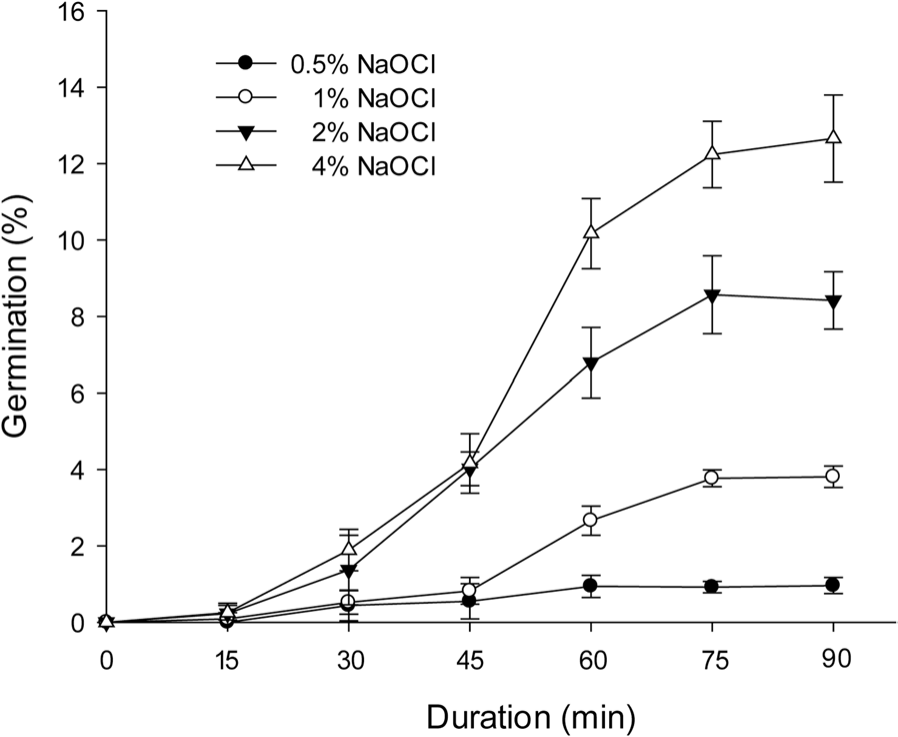
Effect of different concentrations and durations of sodium hypochlorite pretreatments on seed germination in vitro of *V. planifolia*. Data were recorded after 60 days of culture. Data are mean ± SD (n = 3)

Pretreatment with sodium hypochlorite solutions remarkably breaks the situation of no germination of *V. planifolia* mature seeds under the asymbiotic germination treatments. Seeds collected at 135 DAP were soaked with a 2% sodium hypochlorite solution over 60 min before the asymbiotic germination cultures. The pretreatment with sodium hypochlorite solutions resulted in over 5% seed germination (Fig. 3). The effect of sodium hypochlorite soaking treatment on *V. planifolia* seed germination increased with higher strength of sodium hypochlorite and longer soaking time. However, the effect of soaking time reached a maximum at 75 min in all of the different concentrations of sodium hypochlorite solutions (Fig. 3). In addition, *V. planifolia* mature seeds treated with the 4% sodium hypochlorite solution for 75 min can reach over 10% seed germination.

### Development of pod, embryo and seed coat

**Fig. 4:**
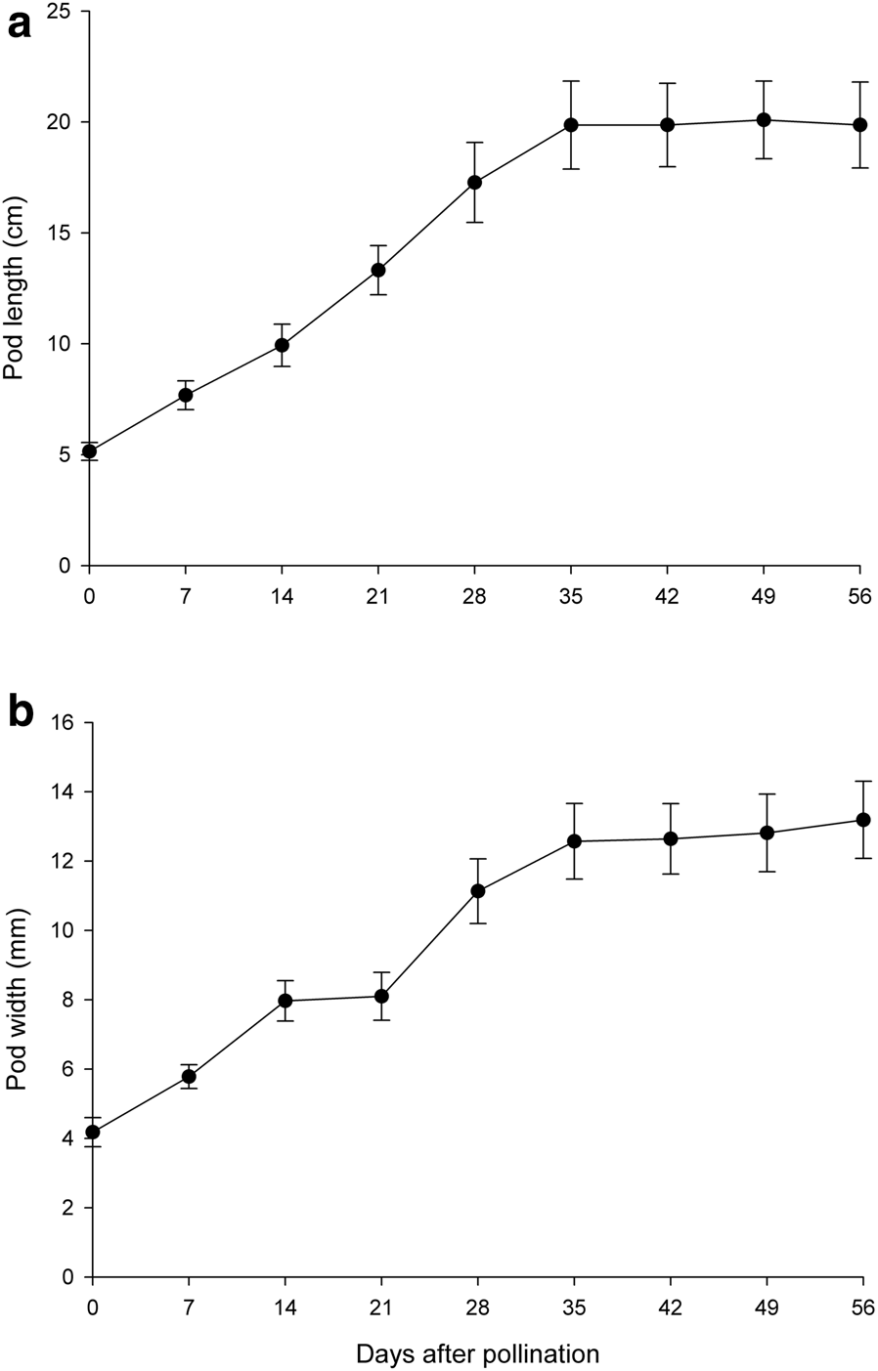
Changes in pod length (**a**) and width (**b**) of *V. planifolia*. Data are mean ± SD (n = 30)

The main structural changes occurring within developing pods from anthesis until maturity are summarized in Table 1. Detailed characteristics are described in follows. Pods are developed from ovaries. After successful hand-pollination, ovaries began to enlarge and elongate rapidly (Figs. 1 and 4). The pod length increased steadily and reached the maximum size (19.86 ± 1.99 cm) at 35 DAP, while the diameter of the pod reached the maximum size (12.57 ± 1.09 mm) at 49 DAP (Fig. 4). By 240 DAP, the pod matured and became yellow, then turned black after 300 DAP (Fig. 1d).

**Table 1:** Major microscopic structural events occurring in the developing pods of *Vanilla planifolia* after hand pollination

DAP	Developmental stage	Seed color
30	Fertilization and the formation of zygote	White
45	Proembryo	A mixture of white and brown seeds
60	Proembryo and the developing of early globular embryo	Most seeds turned black
75	Early globular embryo	Black
90	Globular embryo	Black
105	Late globular embryo	Black
300	Pod ripe and split^a^	Black

^a^From 105 to 300 DAP, the seed structure did not change, but the pod gradually became ripe and turned yellow by 240 DAP

*DAP*  days after pollination

**Fig. 5:**
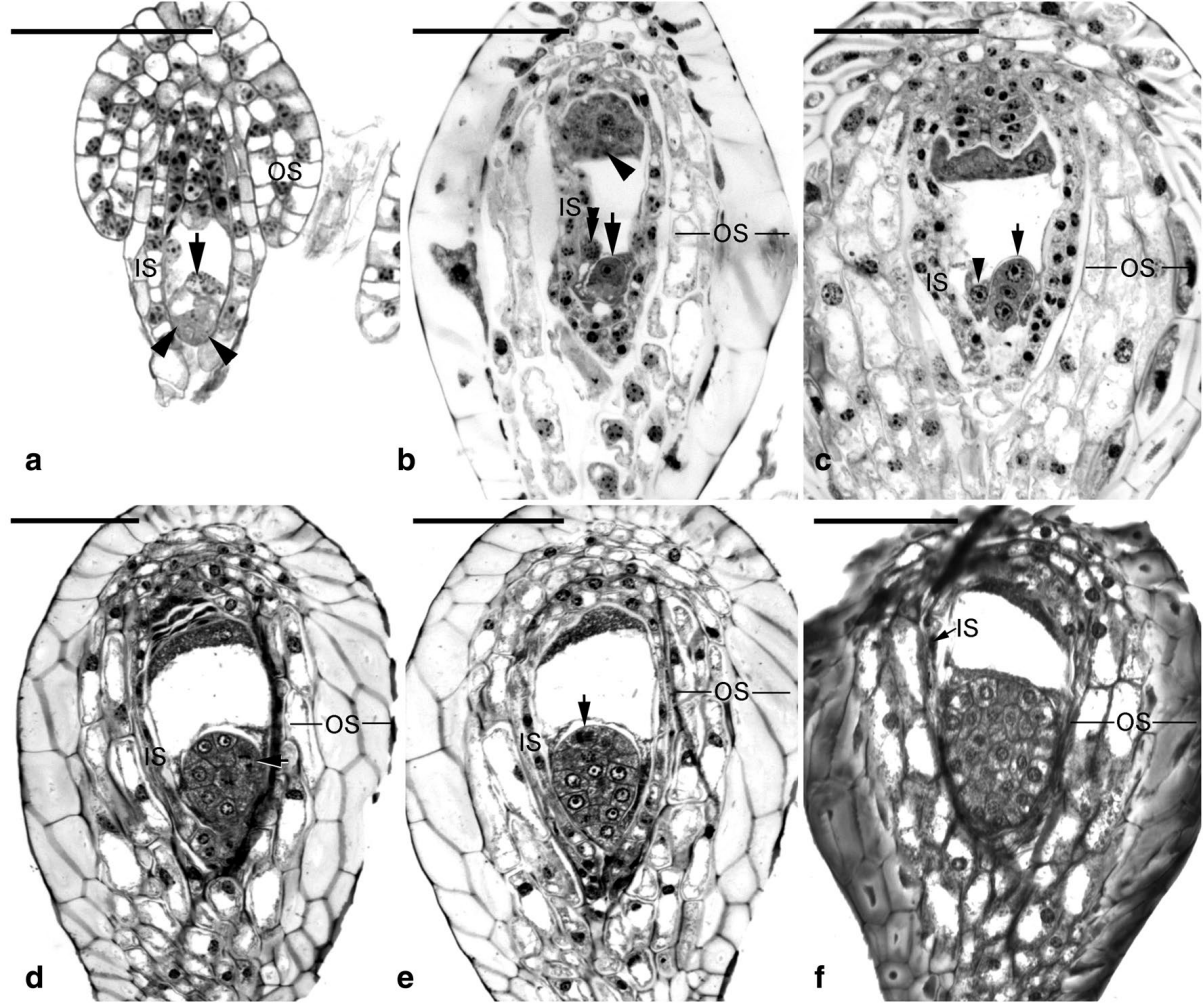
The early embryo development of *V. planifolia*. **a** A mature embryo sac showing the egg apparatus, including the egg cell (arrow) and two synergids (arrowheads). The outer seed coat (OS) was elongating and had not enclosed the inner seed coat (IS). **b** At 30 DAP, after fertilization, zygote (arrow) had a dense cytoplasm, and the degenerated antipodal cells (arrowhead) at the chalazal end were densely stained. In this species, the endosperm failed to develop, and a degenerated endosperm nucleus (double arrowhead) could be observed. **c** Light micrograph showing a three-celled proembryo (arrow), and a degenerated endosperm nucleus (arrowhead) stayed beside the zygote. **d** By 45 DAP, an anticlinal division (arrow) occurred in the outmost cell layer, resulting in the formation of the globular-shape embryo. **e** Light micrograph showing an early globular embryo with an additional anticlinal division (arrow). This species did not have a distinct suspensor during embryo development. **f** Light micrograph showing a longitudinal section through a globular embryo at 60 DAP. The outer most layer of outer seed coat (OS) became lignified. Scale bar = 100 μm

**Fig. 6:**
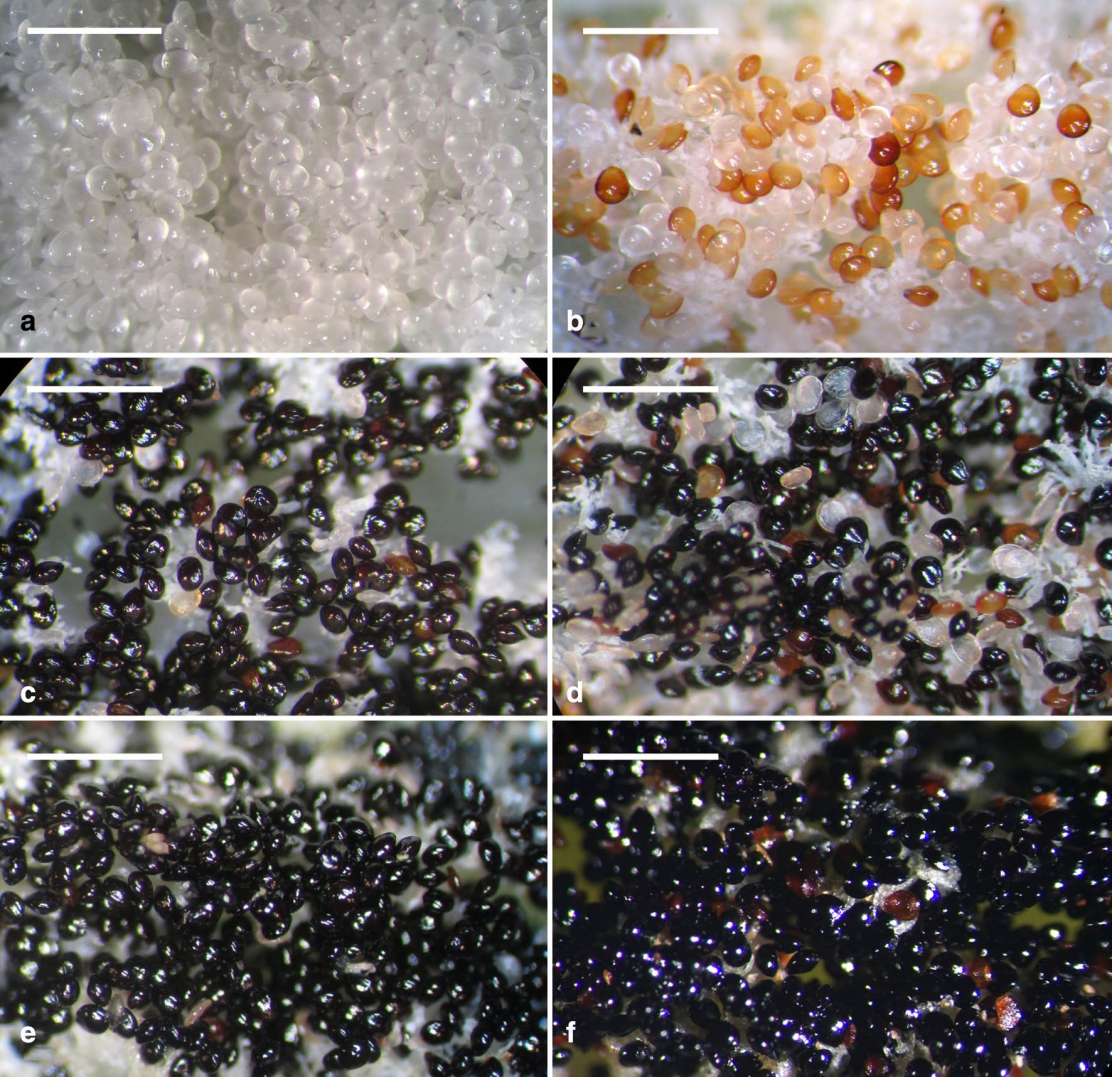
Morphology of developing seeds of *V. planifolia*. **a** White and moist seeds at 30 DAP. **b** A mixture of white and brown seeds at 45 DAP. **cc** A number of seeds had turned black at 60 DAP. **d** Nearly all seeds turned black at 75 DAP. **e** Black seeds at 90 DAP. **f** Black seeds at105 DAP. Scale bar = 1 mm

**Fig. 7:**
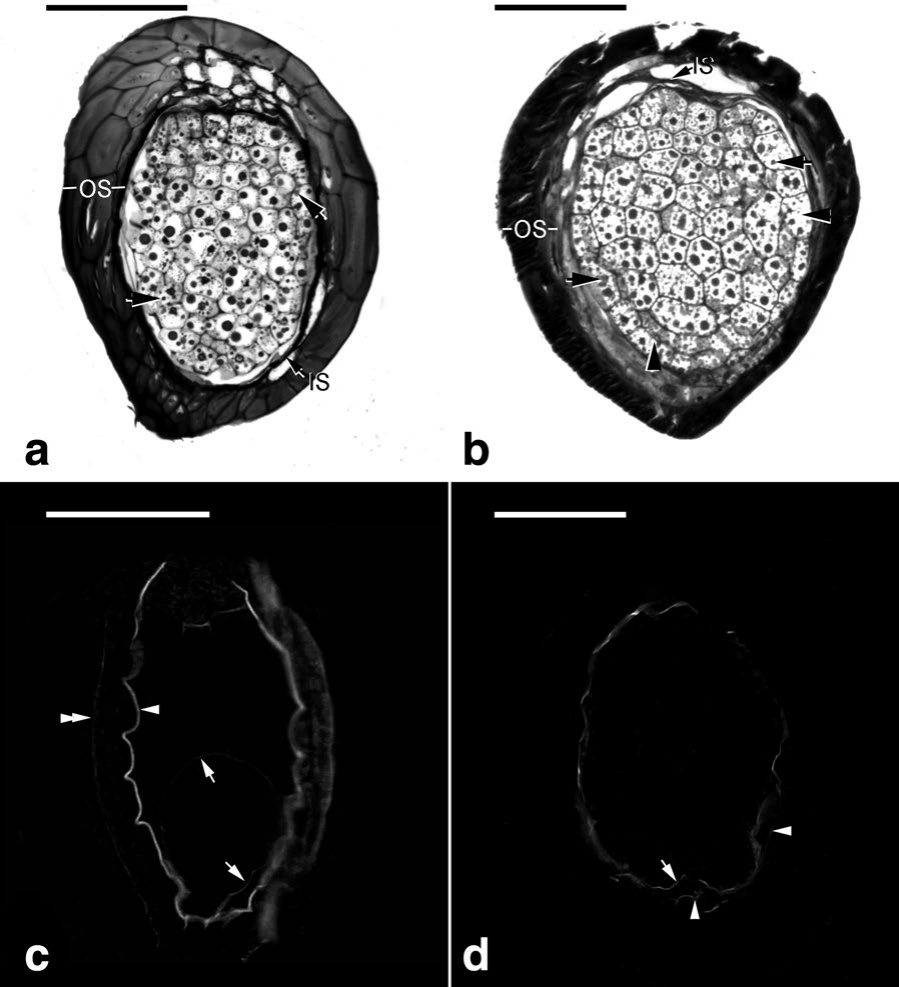
The late embryo development of *V. planifolia*. **a** As the seed approached maturity, a number of tiny protein bodies (arrows) appeared within the embryo proper cells after amido black 10B stain. The thickened outer seed coat (OS) became dehydrated and compressed, with the inner seed coat (IS) compressed into a thin layer. **b** Light micrograph showing a longitudinal section through a mature seed. Several tiny protein bodies (arrows) were found within the embryo proper cells. In this preparation, the lipid bodies were not preserved; the spaces (arrowheads) between the protein bodies were occupied by storage lipid bodies. **c** Nile red staining fluorescence micrograph of an early globular embryo at the stage similar to Fig. 3E. After Nile red staining, the surface wall of the embryo proper (arrows) reacted weakly to the stain, and the innermost (arrowhead) and outermost (double arrowhead) walls of the inner seed coat also reacted positively. **d** Nile red staining fluorescence micrograph of a mature seed at the stage similar to Fig. 5B. The inner seed coat compressed into a thin layer and attached the embryo tightly. The thin inner seed coat (IS, arrowheads) and the surface of the embryo proper (arrow) reacted positively to the stain. Fluorescence was never observed in the thickened outer seed coat (OS). Scale bar = 100 μm

Embryos in a pod were developed from zygotes that were combined between sperm cells in pollens and eggs in embryo sacs. The megaspore mother cells develop just after pollination in early-mid May. Numerous mature embryo sacs could be observed in a pod before fertilization. At 30 DAP, zygotes and proembryos were present within developing pods, and no endosperm was observed (Fig. 5b, c). At this stage, the seeds were white and moist (Fig. 6a). At 45 DAP, additional cell divisions occurred within the inner tiers and the surface layer (Fig. 5d, e), thus resulting in the growth of the embryo proper. Some seeds had turned light to dark brown (Fig. 6b). At 60 DAP, more cell divisions occurred within the embryo proper, resulting in an early globular embryo (Fig. 5f). At this stage, most seeds had turned black (Fig. 6c). This species lacked a structurally defined suspensor during embryo development (Figs. 5 and 7). As the embryo developed to the globular stage at 75 DAP, the embryo proper had filled the cavity of the embryo sac (Fig. 7a). After this stage, nearly all seeds were black (Fig. 6d–f). By 105 DAP, the mature embryo was about 11 cells long and 7 cells wide without the formation of shoot apical meristem and cotyledon (Fig. 7b). At this stage, the cytoplasm of the embryo proper cell was filled with a large number of storage products (e.g., protein bodies and lipid bodies), and starch grains had disappeared. By 300 DAP, the pod had fully matured and desiccated. Then, the pod split, and the mature seeds were released (Fig. 1d).

**Fig. 8:**
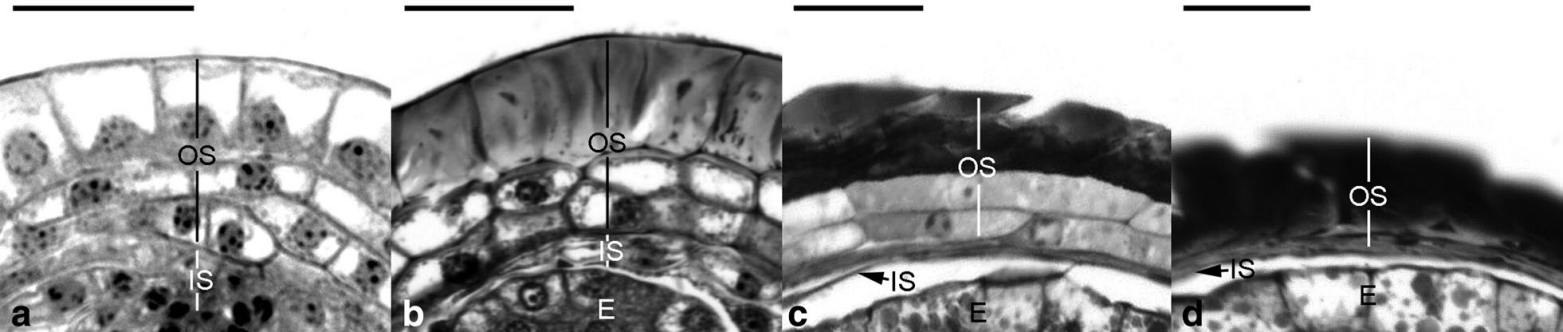
The seed coat development of *V. planifolia*. **a** The seed coat consisted of the inner seed coat (IS, two cells thick) and outer seed coat (OS, three to four cells thick). At the time of fertilization, the cell wall of the outermost layer of outer seed coat still remained primary in nature. **b** At the globular embryo stage, the cell wall of the outermost layer of outer seed coat (OS) had become thickened, and the inner seed coat (IS) was dehydrating and compressing. **c** As the seed approached maturity, the thickened outermost layer of the outer seed coat had dehydrated and compressed, and the inner layers of the outer seed coat were gradually dehydrating and compressing. At this stage, the inner seed coat (IS) had compressed into a thin layer. **d** At maturity, both the thin inner seed coat (IS) and the thickened outer seed coat (OS) compressed and enveloped the embryo (E) tightly. Scale bar = 20 μm

Regarding the development of seed coat, the inner and outer seed coats derived from the inner and outer integuments of the ovule, respectively (Fig. 5a), and these two distinct layers of seed coat surrounded the embryo in the mature seed of *V. planifolia* (Fig. 7b). The inner seed coat was two cells thick, and the cell wall of the inner seed coat remained primary in nature during the early stage of seed development (Figs. 5b, c, 8a). As the seeds approached maturity, the inner seed coat gradually compressed (Figs. 5d–f, 8b), and eventually became a thin layer at maturity (Fig. 8C). However, the outer seed coat consisted of three to four cell layers (Fig. 5b, c). Before fertilization, the outer seed coat was still growing and had not enclosed the embryo sac completely (Fig. 8a). At this stage, the cell wall of the outer seed coat was relatively thin (Fig. 8a), and the cell wall of the outermost layer of the outer seed coat became thickened after fertilization (Fig. 5b). By 60 DAP, the thickened wall of the outermost layer of the outer seed coat became sclerified (Figs. 5f and 8b). As the seed approached maturity, the inner layers of the outer seed coat gradually compressed and attached to the sclerified outermost layer of the outer seed coat (Fig. 8c, d). Using TBO staining, the cell wall of the outermost layer of the outer seed coat stained greenish-blue, indicating the presence of phenolic compounds in the cell wall (Fig. 8b, c). In addition, using Nile red staining, the surface wall of the embryo proper and the innermost and outermost walls of the inner seed coat reacted positively, which suggested the possible accumulation of a cuticular substance in the wall of these two layers (Fig. 7c, d).

### Protocorm and seedling growth

**Fig. 9:**
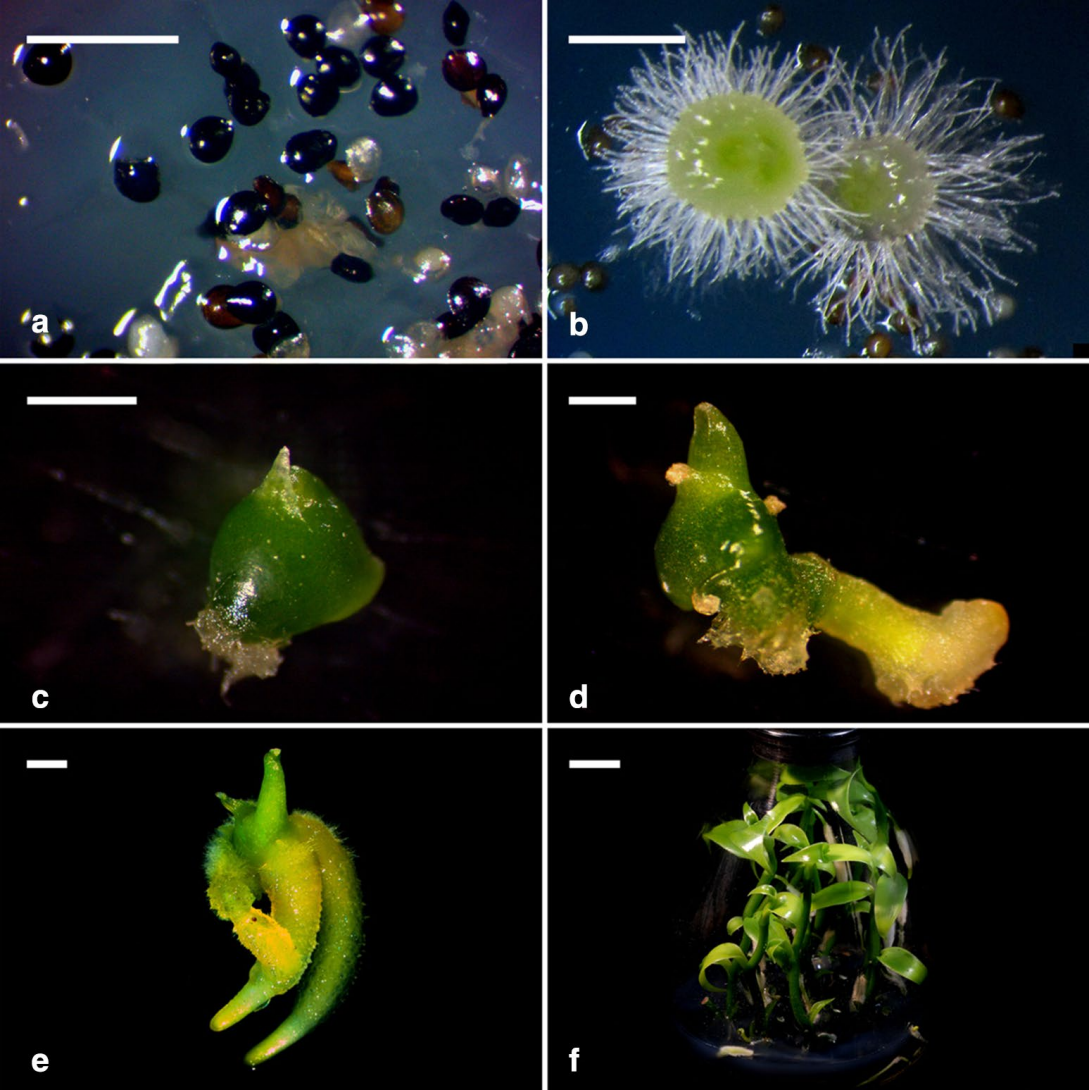
Protocorm development and seedling growth of *V. planifolia*. **a** By 30 days of culture, a few embryos had emerged from the seed coat. Scale bar = 1 mm. **b** Young protocorms with numerous rhizoids appeared at the basal protocorms by 60 days of culture. Scale bar = 1 mm. **c** The protocorm had enlarged with the differentiation of a shoot tip by 90 days of culture. Scale bar = 1 mm. **d** The protocorm had a differentiated first root by 120 days of culture. Scale bar = 1 mm. **e** By 150 days of culture, the protocorm had elongated with the prominent root formation, ready to transfer to the growth medium. Bar = 2 mm. **f** After 90 days of culture on the growth medium, the seedlings grew into vines with several healthy roots. Bar = 2 cm

For the development of protocorms, most embryos were still enveloped by the seed coat at 30 days of culture on 1/2 MS medium. Only a few embryos had emerged from the seed coat, which was considered seed germination (Fig. 9a). By 60 days of culture, young protocorms had turned pale green, and numerous rhizoids appeared at the basal protocorms (Fig. 9b). By 90 days of culture, the protocorm had enlarged and turned dark green to differentiate shoot apical meristem (Fig. 9c). Subsequently, the developing protocorm with the differentiation of the first root was observed (Fig. 9d). By 150 days of culture, the protocorm had further elongated with prominent root formation (Fig. 9e). After transferring onto the seedling growth medium for additional 90 days, the seedlings grew into vines with several healthy roots that could be taken out of flasks (Fig. 9f).

## Discussion

The mature seed of *V. planifolia* is black and hard, which is distinct from the seeds of many orchids (Arditti and Ghani 2000). In the present study, based on a defined time frame, we investigated the morphological and structural changes of seeds (Table 1; Figs. 5, 6 and 7). In many orchids, the outer seed coat develops from two cell layers (Yam et al. 2002), whereas in *V. planifolia*, the outer seed coat is derived from four cell layers (Figs. 5 and 8). The histological and histochemical results indicated that the sclerification primarily occurred in the outermost cell layer of the outer seed coat (Figs. 5, 7 and 8). Similar hard seeds can be found in a few vanilloid species, e.g., *Galeola septentrionalis* (Suetsugu et al. 2015) and *G. javanica* (Yang and Lee 2014). Their seed coats are multi-layer, and the walls are heavily thickened with lignin polymers as seeds mature. The cell wall of the outermost cell layer of the outer seed coat stained greenish-blue by TBO indicated the presence of phenolic compounds (Fig. 8). In the seed coat of many plants, phenolic compounds, including caffeic acid, sinapic acid, coumarin, chlorogenic acid, and ferulic acid, are esterified to the wall structure (Gubler and Ashford 1985; Pan et al. 2002). The accumulation of phenolic compounds in the seed coat inhibits seed germination (Bewley and Black 1994). In *V. planifolia*, further analysis by nuclear magnetic resonance spectroscopy revealed the heavy deposition of catechyl units during lignification of the cell wall of the seed coat (Chen et al. 2012).

Also, the presence of a cuticular substance or suberin has been detected in the cell wall of the inner seed coat (also known as a carapace) and/or outer seed coat of some orchids, such as *Cephalanthera*, *Cymbidium*, and *Cypripedium* (Carlson 1940; Yeung et al. 1996; Lee et al. 2005; Yamazaki and Miyoshi 2006). In *V. planifolia*, after Nile red staining, a thin fluorescent layer was observed in the innermost layer of the inner seed coat cells and the surface wall of the globular embryo, which suggests the accumulation of cuticular substance in walls. However, the fluorescence pattern was absent in the walls of the outer seed coat (Fig. 7c, d). In orchids, the deposition of cuticular substance on the surface of the embryo proper and in the innermost layer of the seed coat may provide physical protection and ensure moisture retention to developing embryos.

Asymbiotic germination has been reported to support the growth of the immature embryo in several terrestrial orchid species (Lee et al. 2005, 2007; Van Waes and Debergh 1986). In this study, we investigated the effect of seed maturity and seed pretreatments on asymbiotic germination. The optimal germination was observed at 45 DAP (Fig. 3). At this stage, the early globular embryo appeared, and the outermost cell layer of the outer seed coat had become thickened but had not been heavily lignified (Fig. 5d). *V. planifolia* is an epiphytic orchid species and pantropical in distribution, while its pattern of seed germination is similar to those of terrestrial orchids from temperate regions, where immature seeds are easier to germinate in vitro than are mature seeds (Linden 1980; Zhang et al. 2013). It is also notable that the optimal germination at 45 DAP only reached 9.9%. The very low germination ability in immature seeds of *V. planifolia* could be due to the impermeability of thickened outer seed coat and/or the presence of inhibitors, e.g. ABA or phenolics during the early stage of seed development. The germination decreased sharply by 60 DAP, which agreed with the initiation of seed coat lignification (Fig. 3). Because the outermost cell layer of the outer seed coat had been lignified, a stronger hydrophobic nature was observed during the operation of seed sowing. As the seed matured, the outer seed coat gradually compressed into a thick and heavily lignified layer surrounding the embryo (Fig. 8). The low permeable seed coat restricted the water uptake and solute diffusion, which resulted in poor germination of mature seeds.

Previous reports have indicated that the poor germination of mature seeds in terrestrial orchids from temperate regions may be attributed to the accumulation of chemical inhibitors to germination, such as abscisic acid (ABA) in *Dactylorhyza* and *Epipactis* (van der Kinderen 1987), *Calanthe* (Lee et al. 2007) and *Cypripedium* (Lee et al. 2015) and phenolics in *Cymbidium* (Kako 1976), and/or the formation of an impermeable container in the seed coat that may make an embryo difficult to obtain water and nutrients for germination (Miyoshi and Mii 1998; Lee et al. 2005). Seed pretreatments may release dormancy and enhance germination by changing the physical integrity of seed coverings, allowing the embryo to absorb water and nutrients and to uptake oxygen (Taylor et al. 1998). Seed pretreatments using bleaching solutions, such as calcium hypochlorite and sodium hypochlorite, can greatly stimulate seed germination of several European terrestrial orchids (Linden 1980; Rasmussen 1995; Steele 1996; Van Waes and Debergh 1986). In this study, the germination of mature seeds was enhanced with soaking in 2 and 4% sodium hypochlorite solutions from 75 to 90 min. However, no germination was recorded in fully mature seeds (Fig. 2). Common bleaching agents such as sodium hypochlorite have been widely used for removing residual lignin from the wood pulp (Holik 2006). From the scanning electron microscopic observations, the seed coat was scarred with hypochlorite oxidation, so the seed coat was relatively more permeable to water (Lee 2011). Of note, as compared with previous reports of seed pretreatments using hypochlorite solutions, we used a stronger concentration (4% sodium hypochlorite) and a longer duration (90 min) to pretreat mature seeds. However, the optimal germination of 12.66 ± 1.14% was relatively low. This low germination may reflect the hard seed coat of *V. planifolia* with its extremely impermeable nature.

In addition to the physical dormancy, the low germination of pretreated seeds may be attributed to the accumulation of inhibitory substances, such as ABA inside the embryo, which still cannot be leached out by hypochlorite solutions. The orchid embryo may be highly sensitive to the presence of endogenous ABA and possess deep physiological dormancy (Lee et al. 2015). Furthermore, the black seed color of *V. planifolia* may be due to the accumulation of phytomelanins (Nishimura and Yukawa 2010), which has a high molecular weight and is formed through the process of oxidation and polymerization of phenols (Glagoleva et al. 2020). In our seed pretreatment experiments, the seed coat remained dark black after soaking with strong hypochlorite solutions. Phytomelanins are known to provide additional mechanical strength to seed coats, protecting the embryos from damage, but phytomelanins can affect seed dormancy and inhibit seed germination (Debeaujon et al. 2000; Gu et al. 2011). In *Cyrtosia* and *Vanilla* species, as their fruits ripen, they usually have vivid colors and/or a heady fragrance to attract animals, such as rats, bats, or birds (Soto Arenas and Dressler 2010; Yang and Lee 2014; Suetsugu et al. 2015). The orchid seed with a heavily lignified, hard seed coat is adapted to the seed dispersal strategy involved in endozoochory by animals (Suetsugu 2018). The thickened lignified seed coat with the accumulation of phytomelanins can protect the embryos when the seeds pass through the digestive tract of animals.

## Conclusions

In this report, we provide reliable protocols for seedling production of *V. planifolia* through asymbiotic seed germination. For the successful culture of immature seeds, the timing of seed collection is short and critical, and optimal germination could be obtained from immature seeds collected at 45 DAP (before seeds turned black). For improving the germination of mature seeds, pretreatment with 4% sodium hypochlorite solution for 75 to 90 min is recommended. We propose that the thickened and lignified seed coat, forming an impermeable envelope, is responsible for the seed coat-imposed dormancy. Further work is needed to examine the changes in levels of endogenous inhibitors in embryos, such as ABA, during seed development. Such studies should help to fully explain the low germination percentage of *V. planifolia* seeds.

## Data Availability

Not applicable.
